# The Environmental Estrogen Bisphenol A Inhibits Estradiol-Induced Hippocampal Synaptogenesis

**DOI:** 10.1289/ehp.7633

**Published:** 2005-02-24

**Authors:** Neil J. MacLusky, Tibor Hajszan, Csaba Leranth

**Affiliations:** ^1^Center for Neural Recovery and Rehabilitation Research, Helen Hayes Hospital, New York, New York, USA; ^2^Department of Obstetrics, Gynecology, and Reproductive Sciences, Yale University School of Medicine, New Haven, Connecticut, USA; ^3^Laboratory of Molecular Neurobiology, Biological Research Center, Hungarian Academy of Sciences, Szeged, Hungary; ^4^Department of Neurobiology, Yale University School of Medicine, New Haven, Connecticut, USA

**Keywords:** bisphenol A, CA1, estradiol, hippocampus, spine synapse density

## Abstract

Bisphenol A (BPA) is an estrogenic chemical that is widely used in the manufacture of plastics and epoxy resins. Because BPA leaches out of plastic food and drink containers, as well as the BPA-containing plastics used in dental prostheses and sealants, considerable potential exists for human exposure to this compound. In this article we show that treatment of ovariectomized rats with BPA dose-dependently inhibits the estrogen-induced formation of dendritic spine synapses on pyramidal neurons in the CA1 area of the hippocampus. Significant inhibitory effects of BPA were observed at a dose of only 40 μg/kg, below the current U.S. Environmental Protection Agency reference daily limit for human exposure. Because synaptic remodeling has been postulated to contribute to the rapid effects of estrogen on hippocampus-dependent memory, these data suggest that environmental BPA exposure may interfere with the development and expression of normal sex differences in cognitive function, via inhibition of estrogen-dependent hippocampal synapse formation. It may also exacerbate the impairment of hippocampal function observed during normal aging, as endogenous estrogen production declines.

Natural and man-made chemicals in the environment can exert hormone mimetic or antagonist activity. Bisphenol A (BPA), a widely used chemical with mixed estrogen agonist/antagonist properties, is employed in the manufacture of plastics used in dental prostheses and sealants (Suzuki et al. 2000), in the linings of metal cans used to preserve foods (Kang et al. 2003), and in such items as baby bottles (Brede et al. 2003) and the clear plastic cages used in many research institutions to house laboratory animals (Howdeshell et al. 2003). The low affinity of BPA for the cell nuclear estrogen receptors ER-α and ER-β and weak bioactivity in standard tests of estrogenicity, such as the rat uterotrophic assay (Ashby 2001), have led to the conclusion that human BPA exposure is probably insufficient to elicit significant estrogenic responses [Degen et al. 2002; European Commission (EC) Scientific Committee on Food 2002; U.S. Environmental Protection Agency (EPA) 1993]. Whether the endocrine activity of BPA is accurately reflected in such tests, however, remains controversial, because of the potential for tissue and cell-specific estrogen effects (Safe et al. 2002). Of particular concern, several reports have indicated that BPA exposure inhibits sexual differentiation of nonreproductive behaviors, including maze learning behavior (Carr et al. 2003; Farabollini et al. 2002), at doses as much as 1,000-fold lower than those required for stimulation of uterine growth (Ashby 2001). The mechanisms underlying these low-dose effects remain unknown.

Sexual differentiation of the brain is believed to involve neurotrophic effects of estrogens, mediated at least in part via activation of kinase-dependent signaling cascades (Toran-Allerand et al. 1999). Kinase-associated neuroplastic responses to estrogen are also expressed in adulthood, in the cornu ammonis (CA) pyramidal neurons of the hippocampus (Bi et al. 2001; McEwen 2002). In adult female rats (Woolley and McEwen 1992) as well as nonhuman primates (Leranth et al. 2002), estradiol induces a rapid increase in CA1 pyramidal cell dendritic spine synapse density (PSSD), a response that has been postulated to involve intermediate activation of the mitogen-activated protein (MAP) kinase cascade (Bi et al. 2001). We reasoned that if BPA inhibits sexual differentiation of the rodent brain, there might also be significant interactions between estradiol and BPA with respect to the regulation of hippocampal CA1 PSSD. Consistent with this hypothesis, in rat hippocampal organotypic cultures, regulation of NMDA receptors, which are critical components of the mechanisms responsible for estrogen regulation of CA1 dendritic spine density (Woolley and McEwen 1994), has been reported to be sensitive to nanomolar concentrations of either 17β -estradiol (E_2_) or BPA (Sato et al. 2002). Therefore, in the present study, we examined the effects of estradiol and BPA, alone and in combination, on CA1 PSSD in adult ovariectomized (OVX) rats. Our results indicate that BPA does indeed have potent effects on the regulation of CA1 PSSD. However, the data demonstrate that, rather than inducing estrogen-like responses, BPA antagonizes the rapid inductive effects of estrogen on hippocampal PSSD.

## Materials and Methods

### Animals.

Experimental protocols were approved by the Institutional Animal Care and Use Committee of Yale University, where all studies using animals were performed. Adult female Sprague-Dawley rats (250–300 g; Charles River Laboratories, Wilmington, MA, USA) were used. The rats were ovariectomized under ketamine/xylazine/acepromazine anesthesia (3 mL/kg intramuscular injection of a cocktail containing 25 mg ketamine, 1.2 mg xylazine, and 0.03 mg acepromazine in 1 mL saline).

### Morphologic studies.

One week after ovariectomy, animals were treated with estrogen, using groups of three animals per treatment condition. In the first PSSD study, 15 rats (five groups of three rats) were injected subcutaneously with either 17β -E_2_ (60 μg/kg; 12 rats) or the sesame oil vehicle (200 μL; three rats). Nine of the 12 estradiol-treated animals were treated simultaneously with increasing doses (40, 120, and 400 μg/kg) of BPA (> 99% purity; Sigma-Aldrich, St. Louis, MO, USA). In the second PSSD experiment, 12 rats were injected subcutaneously (three rats per treatment) with 17α -E_2_ (45 μg/kg), BPA (300 μg/kg), a combination of 17α -E_2_ (45 μg/kg) plus BPA (300 μg/kg), or the sesame oil vehicle (200 μL) alone. Thirty minutes after injection, animals were sacrificed under deep ether anesthesia by transcardial perfusion of heparinized saline followed by a fixative containing 4% paraformaldehyde and 0.1% glutaraldehyde in 0.1 M phosphate buffer (pH 7.35). The brains were removed and postfixed overnight in the same fixative without glutaraldehyde. The hippocampi were then dissected out, and 100 μm vibratome sections were cut perpendicular to the longitudinal axis of the hippocampus. The approximately 90 vibratome sections were divided into 10 subgroups using systematic random sampling and were flat-embedded in Araldite (Electron Microscopy Sciences, Fort Washington, PA, USA).

To correct for processing-induced changes in the volume of the tissue, we calculated a correction factor assuming that the treatments did not alter the total number of pyramidal cells. In all hippocampi, we examined six or seven disector pairs (pairs of adjacent 2-μm semi-thin sections mounted on slides and stained with toluidine blue). We calculated a pyramidal cell density value (*D*) using the formula *D* = *N*/*sT*, where *N* is the mean disector score across all sampling windows, *T* is the thickness of the sections (2 μm), and *s* is the length of the window. Based on these values, a dimensionless volume correction factor *k*_v_ was introduced: *k*_v_ = *D*/*D*_1_, where *D*_1_ is the mean cell density across the groups of hippocampi (Rusakov et al. 1997).

To exclude the possibility that alterations in PSSD might be a consequence of changes in the volume of reference, we used a subset of the vibratome sections for volume estimation of the stratum radiatum of CA1, using the Cavalieri’s principle (Gundersen and Jensen 1987). Areas of CA1 stratum radiatum were measured in each section using Scion Image software (Scion Corp., Frederick, MD, USA), and the total volume of CA1 stratum radiatum in each rat was estimated as


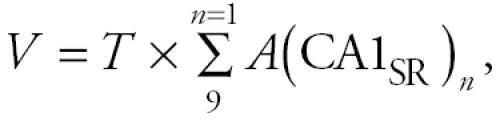


where *T* is the distance between the top of one sampled section and the top of the next section, and *A*(CA1_SR_)*_n_* is the measured area of CA1 stratum radiatum for each section.

Thereafter, serial ultrathin sections were cut from randomly sampled vibratome sections and collected on formvar-coated single-slot grids. Disector pairs of digitized electron micrographs (“reference” and “look-up”) were taken from adjacent ultrasections at a magnification of 11,000× in a Tecnai 12 transmission electron microscope (FEI Company, Hillsboro, OR, USA) furnished with an AMT Advantage 4.00 HR/HR-B CCD camera system (Hamamatsu Photonics, Hamamatsu, Japan), from an area located between the upper and middle third of the CA1 stratum radiatum (300–500 μm from the pyramidal cell layer; Leranth et al. 2004). Identical regions in adjacent sections were identified using landmarks such as myelinated fibers, large dendrites, or blood vessels that did not change significantly between neighboring sections. The investigator taking the electron micrographs was blinded to the treatment of individual animals. Areas occupied by potentially interfering structures such as blood vessels, large dendrites, or glial cells were subtracted from the measured fields. The digitized electron micrographs were printed out using a laser printer and coded. The code was not broken until the analysis was completed. Synapses were counted using a two-dimensional unbiased counting frame with an area of 79 μm^2^ superimposed on the electron microscopic prints. Only those spine synapses were counted that were present in the reference micrograph but not in the look-up micrograph, and vice versa. At least 10 disector pairs were photographed on each electron microscopic grid. With at least three grids (containing two adjacent ultrathin sections) prepared from each vibratome section (cut from three different regions of the hippocampus along its longitudinal septotemporal axis), each animal provided at least 3 × 3 × 10 × 2 = 180 neuropil fields for evaluation. The density of spine synapses in each animal was calculated as


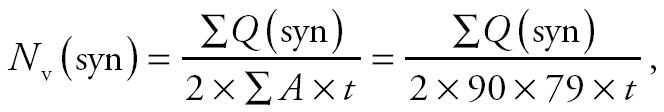


where ∑*Q*(syn) is the total number of synapses sampled by the disector; 2 × 90 = 180 is the number of evaluated electron micrographs per animal; the section thickness *t* was measured by the method of Small’s smallest fold (Weibel 1979; average 0.075 μm); and 79 is the area of the counting frame in square micrometers. PSSD for each animal was calculated by dividing *N*_v_(syn) by the volume correction factor *k*_v_.

### Rat uterine weight assay.

To assess uterotrophic responses, a separate group of 12 rats (four groups of 3) was treated 1 week after ovariectomy with subcutaneous injections of 17β -E_2_ (60 μg/kg), BPA (400 μg/kg), or the combination of BPA (400 μg/kg) and 17β -E_2_ (60 μg/kg), daily for 3 days. Control animals received the sesame oil vehicle (200 μL/day) alone. Six hours after the last injection, the animals were sacrificed; their uteri were dissected free of adhering fat and connective tissue, drained of intraluminal fluid, and weighed.

### Statistical analysis.

Results, in all cases, are presented as mean ± SD of observations from three animals per treatment group. We have verified that the use of three animals per treatment group provides sufficient statistical power to detect effects of the magnitude typically observed after steroid replacement, because of the precision obtained by analyzing large numbers of sections per animal. SDs for counting CA1 synapses in this laboratory are usually < 5% of mean PSSD. With an SD of 5% and sample sizes of three per group, a 15% change in mean PSSD can be detected with α = 0.05 and 80% power. In the present studies, SDs for measurement of PSSD were in most instances considerably < 5% of mean.

Data were analyzed statistically using Statview (SAS Institute, Cary, NC, USA) or SPSS for Windows (Systat Inc., Chicago, IL, USA). We used Bartlett’s test to verify homogeneity of variance. One- and two-way analysis of variance (ANOVA) and the Bonferroni-Dunn test were used for comparison of individual treatment groups. When only control versus treatment comparisons were considered, we used the Student *t*-test. Four-parameter least-squares regression analysis of the BPA dose–response data was performed using ALLFIT (DeLean et al. 1978).

## Results

**Figure 1:**
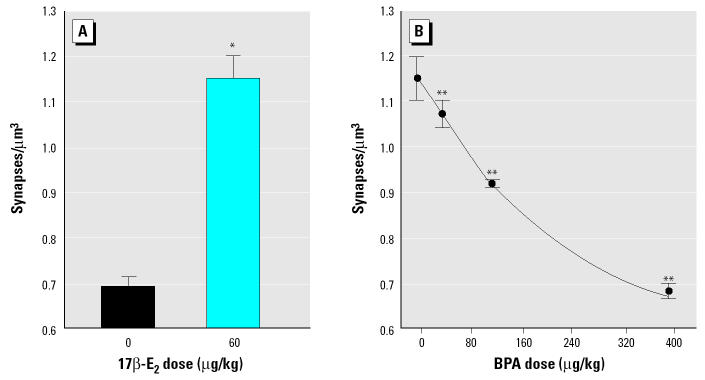
BPA inhibits the effect of 17β -E_2_ on CA1 PSSD. (*A*) At 30 min after 17β -E_2_ injection, PSSD increased by almost 100%. (*B*) The PSSD response to 17β -E_2_ is inhibited in a dose-dependent manner by coadministration of BPA. Data in all cases represent mean ± SD of independent observations from three rats at each dose level. In the case of the 120-μg/kg dose, the bars indicating SD are so close to the mean that they are partially obscured by the symbol. The line through the points represents the four-parameter logistic best-fit regression analysis of the data. The ED_50_ for BPA inhibition of the 17β -estradiol–induced increase in PSSD, calculated from the four-parameter curve fit, is 117 μg/kg.
*Significantly different from oil-injected controls (*p* < 0.001, Student *t*-test).
**Significantly different from PSSD in animals treated with 17β -E_2_ alone (Bonferroni-Dunn post hoc test, *p* < 0.05).

**Figure 2:**
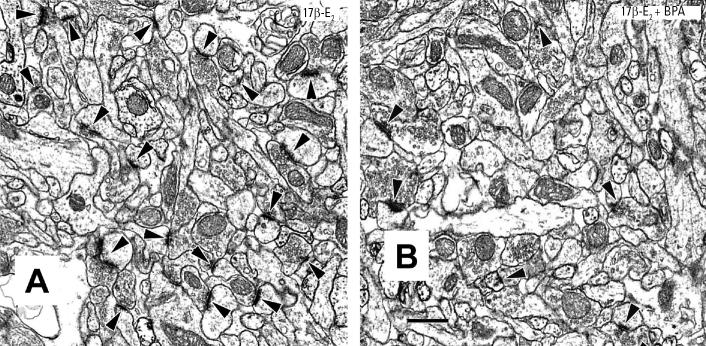
High-power electron micrographs taken from the CA1 stratum radiatum of rats treated with either (*A*) 60 μg/kg 17β -E_2_ or (*B*) 60 μg/kg 17β -E_2_ + 400 μg/kg BPA*.* Arrows indicate spine synapses. Bar = 500 nm.

Treatment of OVX rats with 17β -E_2_ (60 μg/kg body weight) increased CA1 PSSD almost 2-fold (Figure 1A). This dose of 17β -E_2_ has previously been shown to induce a maximal PSSD response (MacLusky et al. 2005). Treatment with BPA did not further enhance hippocampal synapse formation but dose-dependently inhibited the effect of 17β -E_2_ (Figures 1B, 2). At a BPA dose of 400 μg/kg, the PSSD response to 17β -E_2_ was completely inhibited, compared with the CA1 PSSD in OVX vehicle-treated animals. Four-parameter least-squares regression analysis (DeLean et al. 1978) determined a median effective dose (ED_50_) of 117 μg/kg for BPA inhibition of the response to 17β -E_2_.

**Figure 3:**
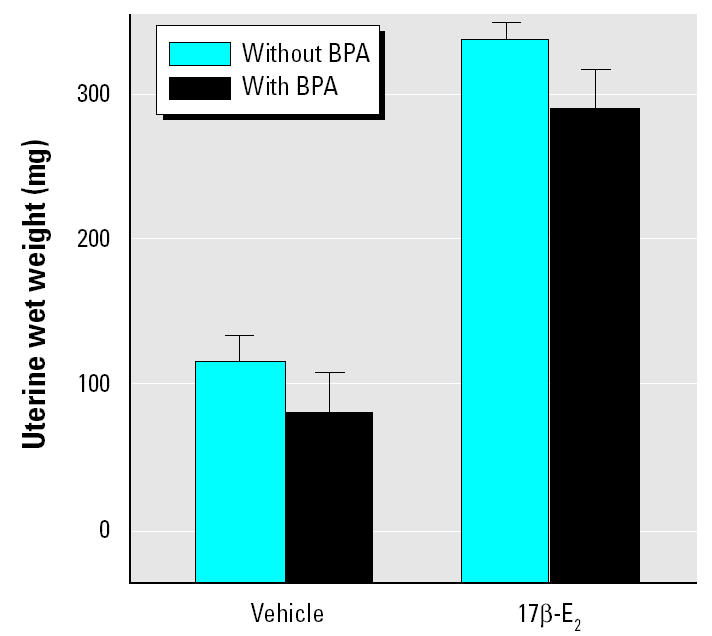
BPA administration only slightly impairs the uterotrophic response to 17β -E_2_. Two-way ANOVA: 17β -E_2_ effect, *F* = 301.2, df 1,8, *p* < 0.0001; BPA effect, *F* = 11.1, df 1,8, *p* = 0.01; 17β -E_2_ × BPA interaction, *F* = 0.29, df 1,8, *p* > 0.6.

Increased uterine weight is a widely accepted bioassay for estrogen action (Ashby 2001). Therefore, we determined whether the dose of BPA (400 μg/kg) found to block induction of PSSD produced comparable inhibition of uterotrophic responses. Administration of the highest dose of BPA daily for 3 days only marginally inhibited the uterotrophic effect of 17β -E_2_ (Figure 3). These results are consistent with previous reports that BPA exerts weak antagonist effects on some uterine responses to estradiol at doses < 100 mg/kg, although it acts as an estrogen agonist at higher dose levels (Ashby 2001).

**Figure 4:**
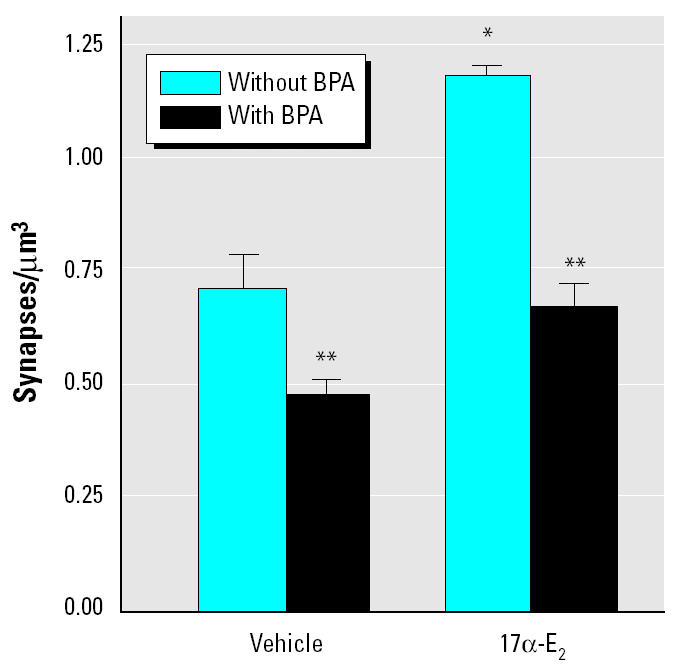
BPA inhibits the effects of 17α -E_2_ on CA1 PSSD. In the absence of BPA, 17α -E_2_ induced an increase in synapse density of 0.463 synapses/μm^3^, a 65% increase above the mean synapse density in vehicle-injected controls. In the presence of BPA, the effect of the estrogen was reduced to an increase of 0.192 synapses/μm^3^, 39% above the level observed in animals treated with BPA alone. Two-way ANOVA: 17α -E_2_ effect, *F* = 237.3, df 1,8, *p* < 0.0001; BPA effect, *F* = 292.8, df 1,8, *p* < 0.0001; 17α -E_2_ × BPA interaction, *F* = 40.8, df 1,8, *p* = 0.0002.
*Significantly higher PSSD than in vehicle-treated rats, without BPA (Student *t*-test, *p* < 0.05).
**Significant inhibitory effect of BPA compared with animals given the same vehicle or 17α -E_2_ without BPA (Bonferroni-Dunn post hoc test, *p* < 0.05).

Like several other responses of neurons to estrogen (Green et al. 1997; Levin-Allerhand et al. 2002; Yu et al. 2004), PSSD is sensitive to both the 17α and 17β isomers of estradiol, the 17α isomer being considerably more potent as an inducer of CA1 spine synapses (MacLusky et al. 2005), despite the fact that it has very low uterotrophic activity (Lundeen et al. 1997). We therefore determined whether BPA also interferes with the synaptic effects of 17α -E_2_. Treatment with 17α -E_2_ at 45 μg/kg induced an increase in PSSD similar to that elicited by 60 μg/kg 17β -E_2_ (Figure 4; compare with Figure 1). Administration of 300 μg/kg BPA alone significantly reduced PSSD. The same dose of BPA also inhibited the increase in PSSD induced by 17α -E_2_ (Figure 4). The mean PSSD observed after treatment with the combination of BPA and 17α -E_2_ was not significantly different from that observed in OVX vehicle-injected controls.

In neither of the two PSSD studies was there any significant variation in the total volume of the CA1 stratum radiatum (Table 1), confirming the validity of the volume correction procedure used in calculating PSSD.

## Discussion

Our data indicate that low-dose BPA exposure inhibits the rapid hippocampal synaptogenic response to estradiol. The minimum BPA dose required to elicit this effect is within the range of dose levels believed, until now, to have little or no significant biologic impact, even under conditions of long-term BPA exposure. In the United States, the current U.S. Environmental Protection Agency (EPA) maximum acceptable “reference” dose for chronic BPA ingestion is 50 μg/kg /day, calculated as 0.1% of the lowest observed adverse effect level (LOAEL) determined from toxicity studies (U.S. EPA 1993). The corresponding European Commission tolerable daily intake (TDI; 10 μg/kg/day) is based on the assumption of a 500-fold safety margin over the no observed effect level (NOEL) dose derived from three-generation rat reproductive toxicity trials (EC Scientific Committee on Food 2002). In OVX rats, our data indicate that a single BPA dose of 40 μg/kg, below the U.S. EPA reference dose and only 4-fold higher than the European Commission TDI safe daily limit, is sufficient to significantly impair the PSSD response to maximal 17β -E_2_ stimulation. Under conditions of low physiologic estrogen exposure, as is the case during prepubertal development as well as after reproductive senescence, considerably lower doses of BPA may be sufficient to interfere with the synaptogenic effects of the hormone. Circumstantial evidence supporting the view that the effects of BPA are not confined to rapid PSSD responses to estrogen administration is provided by the data for OVX rats. Even without estrogen treatment, in OVX rats BPA significantly reduced CA1 PSSD (Figure 4). Preliminary data from our laboratories indicate that the “baseline” PSSD observed in OVX rats includes a contribution from the phytoestrogens present in normal rat chow. Removal of these estrogens, by feeding with phytoestrogen-free chow, reduces CA1 PSSD to levels comparable with those observed in the present study after treatment of OVX animals with BPA (Leranth C, Hajszan T, MacLusky NJ, unpublished data).

Estradiol has important neurotrophic and neuroprotective functions in the brain, in addition to its role as a reproductive steroid (Nathan et al. 2004). A growing body of evidence indicates that estradiol is synthesized in the hippocampus (Hojo et al. 2004), providing a local source of estrogen onto which the effects of circulating levels of the hormone are superimposed. Because synapse formation in the hippocampus is believed to be involved in the mechanisms mediating the acquisition and retention of memory (Silva 2003), interference with estrogen action in the hippocampus could have serious long-term consequences. Deficiencies in gonadal steroid-induced stimulation of hippocampal synaptogenesis have been suggested to contribute to neuro-degenerative disorders and age-related cognitive impairment, for which women with low bioavailable circulating estradiol concentrations appear to be at enhanced risk (Gandy 2003; Yaffe et al. 2000). The ability of BPA to block the effects of estrogen on CA1 PSSD raises the possibility that chronic environmental exposure to BPA might interfere with estrogen effects on the development and function of the brain, inhibiting normal sex differences in nonreproductive behavior (Carr et al. 2003; Farabollini et al. 2002) as well as exacerbating the negative impact on the aging brain of declining gonadal hormone levels (Gandy 2003; Yaffe et al. 2000). Although it remains to be determined whether such effects have a significant impact on the health of human and animal populations exposed to BPA, the current exposure limits clearly do not provide a wide margin of safety in terms of the acute estrogen-dependent regulation of CA1 PSSD.

The mechanisms responsible for BPA’s effects remain unknown. BPA interacts with a number of hormone receptor systems, including androgen and thyroid receptors as well as ER-α and ER-β (Moriyama et al. 2002; Wetherill et al. 2002; Zoeller et al. 2005), providing several potential pathways through which BPA could interfere with hippocampal synaptogenesis. Recent work has demonstrated that BPA and 17β -E_2_ are equipotent activators of CREB phosphorylation in pancreatic islet cells, a response mediated via a “nonclassical” membrane ER (Quesada et al. 2002). Although the *in vitro* equilibrium binding affinities of ER-α and ER-β for BPA are low (Kuiper et al. 1998a), this does not preclude the possibility that BPA could act via these ERs as well because rapid membrane ER-mediated responses may not reflect equilibrium binding affinity. ER-α and ER-β both partially localize to the plasma membrane, where they mediate activation of kinase-dependent signaling pathways. Induction of these rapid kinase-mediated mechanisms exhibits a different pharmacologic specificity than do nuclear receptor–activated responses. Thus, activation of extracellular-signal–regulated kinase (ERK) phosphorylation in rat-2 cells transfected with ER-α or ER-β is equally sensitive to 17α -E_2_ and 17β -E_2_ (Wade et al. 2001), despite the large difference that exists between these steroids in nuclear ER-α and ER-β equilibrium binding affinity (Kuiper et al. 1998b) and uterotrophic activity (Lundeen et al. 1997). Studies in bone cells and ER-transfected HeLa cells suggest that rapid membrane receptor–activated responses to estrogen have a much broader ligand specificity than do slower nuclear receptor–mediated transcriptional effects because ER ligand association rates tend to have a much more relaxed structural specificity than do dissociation rates. Therefore, ligands that are incapable of forming stable nuclear ER complexes because they dissociate rapidly from the receptor may, nonetheless, modulate membrane ER-mediated effects (Kousteni et al. 2001).

Circumstantial evidence points to a role for nonclassical receptor mechanisms in the hippocampal response to estrogen. Effects of 17β -E_2_ on CA1 dendritic structure are accompanied by increased ERK phosphorylation (Bi et al. 2001), as well as changes in the distribution of the phosphorylated form of the serine-threonine kinase Akt in CA1 pyramidal cell dendrites (Znamensky et al. 2003). The fact that 17α -E_2_ and 17β -E_2_ both induce an increase in PSSD is consistent with the hypothesis that membrane-associated ERs may mediate rapid estrogen activation of CA1 spine synapse formation (Wade et al. 2001). That the rapid actions of estradiol on CA1 PSSD involve nonclassical ER systems is also suggested by recent data from this laboratory demonstrating that short-term induction of CA1 spine synapses requires relatively high circulating 17β -E_2_ concentrations (MacLusky et al. 2005). The effects of BPA on rapid estrogen induction of CA1 PSSD may reflect interference, directly or indirectly, with this putative novel estrogen response pathway. Such a hypothesis would be consistent with recent studies in *Mytilus* that have demonstrated marked inhibition of p38 MAP kinase phosphorylation by low concentrations of BPA, a response diametrically opposite to that of estradiol (Canesi et al. 2004, 2005). A critical experiment for future studies will be to determine whether the effects on hippocampal PSSD of sustained physiologic circulating levels of 17β -E_2_ (Woolley and McEwen 1992), which may involve a greater contribution from nuclear ER-α and/or ER-β, are similarly affected by low-dose BPA exposure.

In summary, these data demonstrate that the environmental estrogen BPA inhibits estrogen-activated hippocampal spine synapse formation. Because hippocampal spine synapses are believed to be involved in the mechanisms responsible for the formation of memory (Silva 2003), these observations raise concerns regarding the potential impact of low-dose continuous BPA exposure on cognitive development and function. In addition, they further emphasize the dangers inherent in reliance on only one or a few nuclear ER-dependent tests as a basis for environmental estrogen risk assessment (Safe et al. 2002). There may be other compounds in the environment—natural and man-made—that, like 17α -E_2_ and BPA, exert potent effects on neural estrogen response mechanisms, even though their reported affinities for ER-α and ER-β are low. If so, current screening methods for the evaluation of putative estrogen-like “endocrine disruptors” (U.S. EPA 1998) may underestimate the potential risk of exposure to such compounds.

## Figures and Tables

**Table 1:** Effect of 17β -E_2_ or 17α -E_2_ with or without BPA on total CA1 stratum radiatum volume (mean ± SD).

Treatment	CA1 stratum radiatum volume (mm^3^)
17β -E_2_	
Vehicle control	3.84 ± 0.15
60 μg/kg 17β -E_2_	3.99 ± 0.26
60 μg/kg 17β -E_2_ + 40 μg/kg BPA	3.96 ± 0.16
60 μg/kg 17β -E_2_ + 120 μg/kg BPA	4.05 ± 0.49
60 μg/kg 17β -E_2_ + 400 μg/kg BPA	3.93 ± 0.43
17α -E_2_	
Vehicle control	3.97 ± 0.42
45 μg/kg 17α -E_2_	3.97 ± 0.39
300 μg/kg BPA	3.97 ± 0.37
45 μg/kg 17α -E_2_ + 300 μg/kg BPA	3.77 ± 0.19

The volume of CA1 stratum radiatum was measured in the animals used for spine synapse counting (*n* = 3 animals per group). For animals treated with 17β -E_2_ ± BPA, synapse densities are shown in Figure 1; one-way ANOVA: *F* = 0.162, df 4,10, *p* > 0.95. For animals treated with 17α -E_2_ ± BPA, synapse densities are shown in Figure 4; one-way ANOVA: *F* = 0.241, df 3,8, *p* > 0.85.
